# Immune and tumor-intrinsic signatures are associated with resistance and response to PD-1 blockade in cutaneous squamous cell carcinoma

**DOI:** 10.1007/s00262-026-04610-w

**Published:** 2026-10-30

**Authors:** Viola K. DeTemple, Maximilian Beikirch, Alina C. Ulama, Moritz Bergmann, Sabine Bredemeier, Vivien Schacht, Michael Sachse, Peter Mohr, Thilo Gambichler, Ralf Gutzmer, Katrin Schaper-Gerhardt

**Affiliations:** 1 Department of Dermatology, Ruhr-University Bochum, Johannes Wesling Medical Center, Campus Minden, Hans Nolte Str. 1, 32429 Minden, Germany; 2 Department of Dermatology, Allergy and Venerology, Hannover Medical School, Hannover, Germany; 3 Department of Dermatology, Klinikum Bremerhaven, Bremerhaven, Germany; 4 Department of Dermatology, Elbe Kliniken Buxtehude, Buxtehude, Germany; 5 Department of Dermatology, St. Josef-Hospital Bochum, Ruhr-University Bochum, Bochum, Germany; 6 Department of Dermatology, Dortmund Hospital gGmbH, University Witten, Herdecke, Germany

**Keywords:** Cutaneous squamous cell carcinoma, cSCC, PD-1 inhibitor, Cemiplimab, Biomarker

## Abstract

**Background:**

Anti-PD-1 therapy has significantly improved clinical outcomes in patients with advanced cutaneous squamous cell carcinoma (cSCC); however, approximately half of patients fail to respond. This study aimed to investigate molecular mechanisms underlying therapeutic resistance and to identify predictive biomarkers.

**Methods:**

Whole transcriptome profiling was performed using HTG EdgeSeq technology on pre-treatment cSCC samples. Clinical data and treatment outcomes were correlated with gene expression profiles, and differentially expressed genes (DEGs) were analyzed in relation to response. Moreover, promising targets were further analyzed at the protein level via immunofluorescence and immunohistochemistry.

**Results:**

Tumors from patients achieving disease control showed a distinct immune-active transcriptional profile characterized by interferon-associated signaling, enhanced antigen presentation pathways, and cytotoxic immune activity. Consistently, IFN-γ and cytolytic activity signatures were higher in patients with favorable clinical outcomes. CIBERSORTx-based deconvolution demonstrated marked interpatient heterogeneity, with T cells, macrophages, and NK cells representing major components of the immune infiltrate, but no major differences in overall immune cell composition between patients with early and late/no progression. Histopathological assessment revealed a tendency toward higher immune cell infiltration in patients achieving disease control. Notably, elevated gene expression of SLC7A5 and SOX2 was associated with poor response and early progression. Also, at protein level, higher SLC7A5 expression was associated with early progression, whereas SOX2 showed a more heterogeneous pattern, with particularly high expression in a subset of progressive tumors.

**Conclusion:**

Our data provide hypothesis-generating evidence that a combination of immune-related and tumor-intrinsic novel molecular markers is associated with response to anti-PD-1 therapy in advanced cSCC, thereby identifying promising candidate biomarkers, which require validation in larger independent cohorts.

**Supplementary Information:**

The online version contains supplementary material available at https://doi.org/10.1007/s00262-026-04610-w.

## Introduction

Cutaneous squamous cell carcinoma (cSCC) represents the second most common non-melanoma skin cancer worldwide, with a rising incidence particularly in aging populations and immunosuppressed individuals [1, 2]. While most cSCCs are effectively treated with surgical excision, advanced or metastatic cases present significant therapeutic challenges and are associated with poor prognosis [3]. In recent years, immune checkpoint inhibitors, e.g., targeting programmed death receptor 1 (PD-1), have revolutionized the treatment landscape for various malignancies, including skin cancer. Specifically, the PD-1 inhibitors cemiplimab and pembrolizumab have demonstrated robust clinical efficacy in patients with advanced cSCC, leading to their regulatory approval (in 2018 by the Food and Drug Administration in the U.S., in 2019 by the European Medicines Agency in Europe) and integration into clinical practice. Both, in the pivotal registration trials and in several recently published real-world clinical data, PD-1 inhibitors have shown response rates of approximately 50% and a manageable safety profile in patients with advanced cSCC [4–6].

However, not all patients benefit equally and may encounter primary resistance or relapse after initial response. Given the histological and molecular heterogeneity of cSCC tumor tissue, understanding the underlying mechanism of treatment response and resistance is essential to optimizing patient selection and improving outcomes. By analyzing gene expression patterns at the mRNA level and verifying them at protein level, we aimed to identify differentially expressed genes, immune-related signatures and cellular processes linked to PD-1 inhibitor response or resistance in cSCC.

## Material and methods

### Study design and patient cohort

We retrospectively collected clinical data and tumor tissue from patients with locally advanced cSCC (lacSCC) and metastatic cSCC (mcSCC) treated with PD-1 inhibitors between May 2019 and December 2022 from 5 skin cancer centers in Germany (Minden, Bochum, Hannover, Buxtehude, Bremerhaven). Only patients with histopathologically confirmed diagnosis of cSCC with at least one cycle of PD-1 inhibitors were included. Tumor response was evaluated in accordance with Response Evaluation Criteria in Solid Tumors (RECIST V.1.1.). Objective response is defined as complete response (CR) or partial response (PR), whereas disease control additionally includes stable disease (SD). Progressive disease (PD) is considered separately.

Progression free survival (PFS) was calculated from initiation of PD-1 inhibitor therapy to disease progression, and overall survival (OS) from therapy initiation to death. If no event occurred, patients were censored at the last day of follow-up or at death from any cause. Data regarding adverse events were collected and graded based on the National Cancer Institute Common Terminology Criteria for Adverse Events (NCI CTCAE, version 5.0). The study was performed in accordance with the Declaration of Helsinki and approved by the Ethics Committee of Ruhr University Bochum Site Bad Oeynhausen, Germany (protocol code 2021–811; date of approval: June 8, 2021). Patient consent was waived due to retrospective anonymous data collection without any risk for the patient.

### Gene expression analysis

Gene Expression data were obtained from FFPE tumor tissue using the HTG EdgeSeq Whole transcriptome panel consisting of 19.398 genes (HTG Molecular Diagnostics, Inc., Tucson, AZ, USA), as per manufacturer’s instructions. In summary, tumor tissue was sectioned at 5 µm and stained with Hematoxylin and Eosin; localization of tumor material was performed by a dermatologist. Suitable tumor tissue with adjacent immune cell infiltrate was selected and marked with a pen. Marked areas were scraped from a corresponding non-stained 10 µm section using a sterile disposable scalpel and further processed per manufacturer’s instructions. RNA was extracted by HTG Lysis Buffer to subsequently bind to target-specific Nuclease Protection Probes on the HTG EdgeSeq processor. After the quantitative nuclease protection assay, samples were used as a template in individual PCR reactions with specially designed primers for PCR amplification. Non-incorporated primer tags and RNA were removed from the library. Library concentrations were determined by qPCR, and normalized libraries were pooled and sequenced on the Illumina Nextseq 500 platform (Illumina, Inc., San Diego, CA, USA). Raw data FASTQ files were parsed using the HTG EdgeSeq Parser software. The parsed data provided transcript abundancies per sample for the genes included in the HTG Transcriptome Panel, based on the respective amplified NPP (nuclease protection probes). A generated Excel file combined with an annotation file was uploaded into the web-based HTG Reveal software for quality controls. Further analyses were performed with R studio.

### Immunofluorescence

Native 4 μm tissue sections mounted on Histobond+ slides (Paul Marienfeld GmbH & Co.KG, Lauda-Königshofen, Germany) were deparaffinized using xylene and ethanol, followed by antigen retrieval in demasking buffer composed of Tris–EDTA buffer pH 9.0 (10×, Zytomed, Bremerhaven, Germany) and Ampuwa® (Fresenius Kabi, Bad Homburg, Germany) for 20 min at 95 °C in a steam cooker. Slides were then cooled in ice water and washed three times with wash buffer (Dulbecco’s Phosphate-Buffered Saline (DPBS, PAN Biotech, Aidenbach, Germany) (10 ×), Ampuwa®, and Tween® 20 for Molecular Biology, Sigma-Aldrich/Merck, Darmstadt, Germany), before tissue sections were encircled with a hydrophobic barrier. To block nonspecific antibody binding, slides were incubated in a humid chamber with 10% bovine serum albumin (BSA) blocking solution, prepared from MACS® BSA Stock Solution (Merck) and wash buffer, for 45 min. After removing the blocking solution, primary antibody (LAT1/SLC7A5 (E9O4D) Rabbit mAb (Cell Signaling, Cambridge, U.K.)) diluted in Antibody Diluent was applied and incubated overnight at 4 °C in a humid chamber. Slides were then washed again three times with wash buffer. Secondary antibodies diluted in Antibody Diluent (Zytomed) were applied and incubated for 60 min in a humid chamber. Following additional three washing steps, slides were mounted using FluoroMounter containing 4',6-diamidino-2-phenylindole (DAPI, Bio SB, Santa Barbara, CA, U.S.). Images were acquired using the BZ-X810 fluorescence microscope and BZ-X800 Analyzer software (Keyence, Osaka, Japan).

### Immunohistochemistry

FFPE tissue of cSCC was sectioned at 4 µm and sections were mounted on Histobond+ slides (Paul Marienfeld GmbH & Co.KG). Specimens were stained with primary antibody against anti-SOX2 antibody (1:200) (Abcam, Cambridge, U.K.) following detection using the Mouse/Rabbit ImmunoDetector AP ALK Magenta Detection System (Bio SB). Slides were scanned using a automate scanner (Hamamatsu, Shizuoka Pref., Japan). Expression of SLC7A5 and SOX2 (percentage of positive tumor cells, distribution of positive staining, staining intensity) as well as intensity of the immune cell infiltrate (low, intermediate or high) was scored by two dermatologists, who independently reviewed the slides and reached a consensus assessment. To provide an objective digital validation of the semi-quantitative assessment, SOX2 expression was additionally quantified using QuPath digital image analysis. The digitally determined proportion of SOX2-positive tumor area showed good concordance (data not shown) with the semi-quantitative scores, supporting the robustness of the histopathological evaluation.

### Statistical analysis

Descriptive statistics (medians, interquartile ranges, time intervals and percentual ratios) were calculated in Microsoft Excel (Microsoft 365, version 2419).

For differential gene expression analysis, counts of sequenced RNA were imported in R. After normalization using the median ratio method, differential expression analysis was carried out with an FDR of 0.05 and with *p* value correction for multiple testing via the Benjamini–Hochberg procedure. The obtained fold change estimates were shrunk using apeglm. All methods mentioned above reside in the R package DESeq2 [7]. Differentially expressed genes (DEGs) with a Benjamini–Hochberg adjusted *p* value < 0.05 and |lfc|> 1 were regarded as significant. Volcano plots with highlighted DEGs were created using the R package EnhancedVolcano. Heatmaps of normalized counts were created using the R package ComplexHeatmap [8]. The scores were calculated as mean of log-transformed normalized gene reads on a subset of genes/on signature genes. For the cytolytic score, calculation was done on the genes GZMA and PRF1 (based on the description in Rooney et al. [9]). For the IFN-γ-score, calculation was done on an IFN-γ-signature obtained from GSEA (standard name “HALLMARK_INTERFERON_GAMMA_RESPONSE”/systematic name M5913), composed of exactly 200 genes as well as the Signature from Ayers et al. compromising a 10-gene signature [10]. For GO analysis, gene names were mapped to ensemble IDs using the R packages AnnotationHub (https://bioconductor.org/packages/AnnotationHub*)* and ensembldb [11]. Release 111 of the ensemble database was used for mapping. Then, up- and down-regulated DEGs were analyzed separately using the R package clusterProfiler (ontology “biological process,” *p* value correction for multiple testing via the Benjamini–Hochberg procedure, FDR 0.05; [12]). The resulting GO terms were then aggregated using the R package simplifyEnrichment [13]. The blue-yellow color scheme in part of the heatmaps is provided by the R package viridis. Immune cell composition was estimated from bulk tumor transcriptomic data using CIBERSORTx and xCell. Normalized gene expression data from pre-treatment cSCC samples were used as input for the analysis. Cell-type fractions were estimated using the LM22 leukocyte signature matrix, which distinguishes 22 human hematopoietic cell subsets, including B cells, T cell subsets, natural killer cells, monocytes, macrophages, dendritic cells, mast cells, eosinophils, and neutrophils. CIBERSORTx was run in relative mode with batch correction and without quantile normalization. xCell was run with default settings and the results were normalized to one for each sample (the LM22 reference was obtained from https://dviraran.github.io/xCell2refs/references/LM22.xCell2Ref.rds). For downstream visualization and comparison, inferred immune cell fractions were summarized across individual samples and, where indicated, aggregated into broader immune cell categories. Box plots and stacked bar plots were created with GraphPad Prism (version 10.6.1), comparisons were performed with Mann–Whitney-U-Test.

ROC curves for determination of the optimal cutoff were calculated with R studio using the packages ROCit (https://CRAN.R-project.org/package=ROCit) and dplyr. Survival Curves were generated using “ggplot2,” R package version 0.4.9, (https://CRAN.R-project.org/package=survminer).

### Use of large language models

During the preparation of this manuscript, we used the large language model OpenAI ChatGPT (version GPT-4.1) to exclusively assist in the formulation and editing of certain text passages and to support for literature research. All the content was critically reviewed and revised by the authors to ensure accuracy and compliance with academic standards.

## Results

### Patient characteristics

We enrolled 44 cSCC tissue samples collected from 33 patients, who were treated with PD-1 inhibitors from 5 skin cancer centers in Germany (Minden, Hannover, Bochum, Buxtehude, Bremerhaven). Patient and sample characteristics are summarized in Table 1. The median age was 83 years and 72.7% of the patients were male. Nine patients were immunosuppressed due to medication (n=1) or hematological comorbidities (n=8). Most tumors (81.8%) were localized in UV-exposed areas.

**Table 1:** Patient and sample characteristics

			All	LongPFS	ShortPFS
Patients		n(%)	33 (100)	16 (48.5)	17 (51.5)
Age	Median, years (range)		83 (43–99)	81.5 (49–99)	85 (43–94)
	IQR		76–86	74.8–85	78–86
Age over 80y	yes	n(%)	19 (57.6)	8 (50.0)	11 (64.7)
	no	n(%)	14 (42.4)	8 (50.0)	6 (35.3)
Sex	male	n(%)	24 (72.7)	11 (68.8)	13 (76.5)
	female	n(%)	9 (27.3)	5 (31.3)	4 (23.5)
Best response	CR	n(%)	14 (42.4)	12 (75.0)	2 (11.8)
	PR	n(%)	3 (9.1)	1 (6.3)	2 (11.8)
	SD	n(%)	5 (15.2)	2 (12.4)	3 (17.6)
	PD	n(%)	11 (33.3)	1 (6.3)	10 (58.8)
Overall response	R	n(%)	17 (51.5)	13 (81.3)	4 (23.5)
	NR	n(%)	16 (48.5)	3 (18.7)	13 (76.5)
Progression free survival (PFS)	Median, months (range)		3 (0–19)	12.2 (12–19)	2.6 (0–4)
	IQR		1.3–3.8	12.2–15.6	1–3.2
Early progression	no	n(%)	16 (48.5)	16 (100)	0 (0.0)
	yes	n(%)	17 (51.5)	0 (0.0)	17 (100)
Immunosuppression	yes	n(%)	9 (27.3)	3 (18.8)	6 (35.3)
	no	n(%)	23 (69.7)	13 (81.2)	10 (58.8)
	NA	n(%)	1 (3.0)	0 (0.0)	1 (5.9)
Type of immunosuppression	none	n(%)	23 (69.7)	13 (81.3)	10 (58.8)
	NA	n(%)	1 (3.0)	0 (0.0)	1 (5.9)
	Hematological disease	n(%)	8 (24.3)	3 (18.7)	5 (29.4)
	Medication	n(%)	1 (3.0)	0 (0.0)	1 (5.9)
Follow-up time	Median, months (range)		10 (1.7–40)	14.8 (7.7–28)	8 (1.7–40)
	IQR		7.7–15.4	10.4–17.9	3.9–9.4

			All	Long PFS	Short PFS
Samples		n(%)	44 (100)	22 (50.0)	22 (50.0)
Tumor type	primary	n(%)	21 (47.7)	11 (50.0)	10 (45.5)
	relapse	n(%)	18 (40.9)	8 (36.4)	10 (45.5)
	metastasis	n(%)	5 (11.4)	3 (13.6)	2 (9.0)
UV exposure	yes	n(%)	36 (81.8)	18 (81.8)	18 (81.8)
	no	n(%)	8 (18.2)	4 (18.2)	4 (18.2)
Histological grading	G1	n(%)	6 (13.6)	3 (13.6)	3 (13.6)
	G2	n(%)	8 (18.2)	3 (13.6)	5 (22.7)
	G2-G3	n(%)	3 (6.8)	1 (4.5)	2 (9.1)
	G3	n(%)	12 (27.3)	6 (27.3)	6 (27.3)
	G4	n(%)	2 (4.5)	1 (4.5)	1 (4.5)
	cutaneous metastasis	n(%)	7 (15.9)	5 (22.7)	2 (9.1)
	NA	n(%)	6 (13.6)	3 (13.6)	3 (13.6)

CR, Complete response; PR, Partial response; SD, Stable disease; PD, Progressive disease; IQR, Inter quartile range; PFS, Progression free survival; Long PFS, PFS > 1 year; Short PFS, PFS < 1 year; NA, Not available

During a median follow-up time of 10 months, we observed an objective response by RECIST in 51.5% of the patients. In total, 14 patients showed a complete response (CR), 3 patients a partial response (PR), 5 patients a stable disease (SD) and 11 patients progressive disease (PD) as best response.

### Gene expression profiling

To find molecular features or biomarkers, in connection with response to immune checkpoint inhibition (ICI), we conducted a whole transcriptome analysis of cSCC tumor tissue. In total, 25 samples from 20 patients were analyzed, comprising 15 primary tumors, 5 local recurrences, and 5 metastases. After selecting for pre-treatment samples and skin tissue, we had to exclude 5 samples. Due to duplicate samples from the same patient, we additionally excluded 3 samples from further analysis (Suppl. Figure 1). In the final analysis, we included 17 tumor samples from 17 patients.

For DEG analysis, three different comparisons were applied. In particular we compared (i) responders (CR, PR; n=7) to patients with SD or PD (n=10), (ii) patients with disease control (CR, PR, SD; n=11) to patients with PD (n=6) and (iii) additionally, in order to take patients into account, who experienced an early progress within 12 months after treatment, irrespective of their best response, we also divided the cohort in patients with early (n=10) and late/no progression (n=7). Although the three stratifications address related clinical outcomes, they capture distinct biological dimensions of response. Accordingly, different transcriptional programs dominated the respective comparisons.

**Fig. 1:**
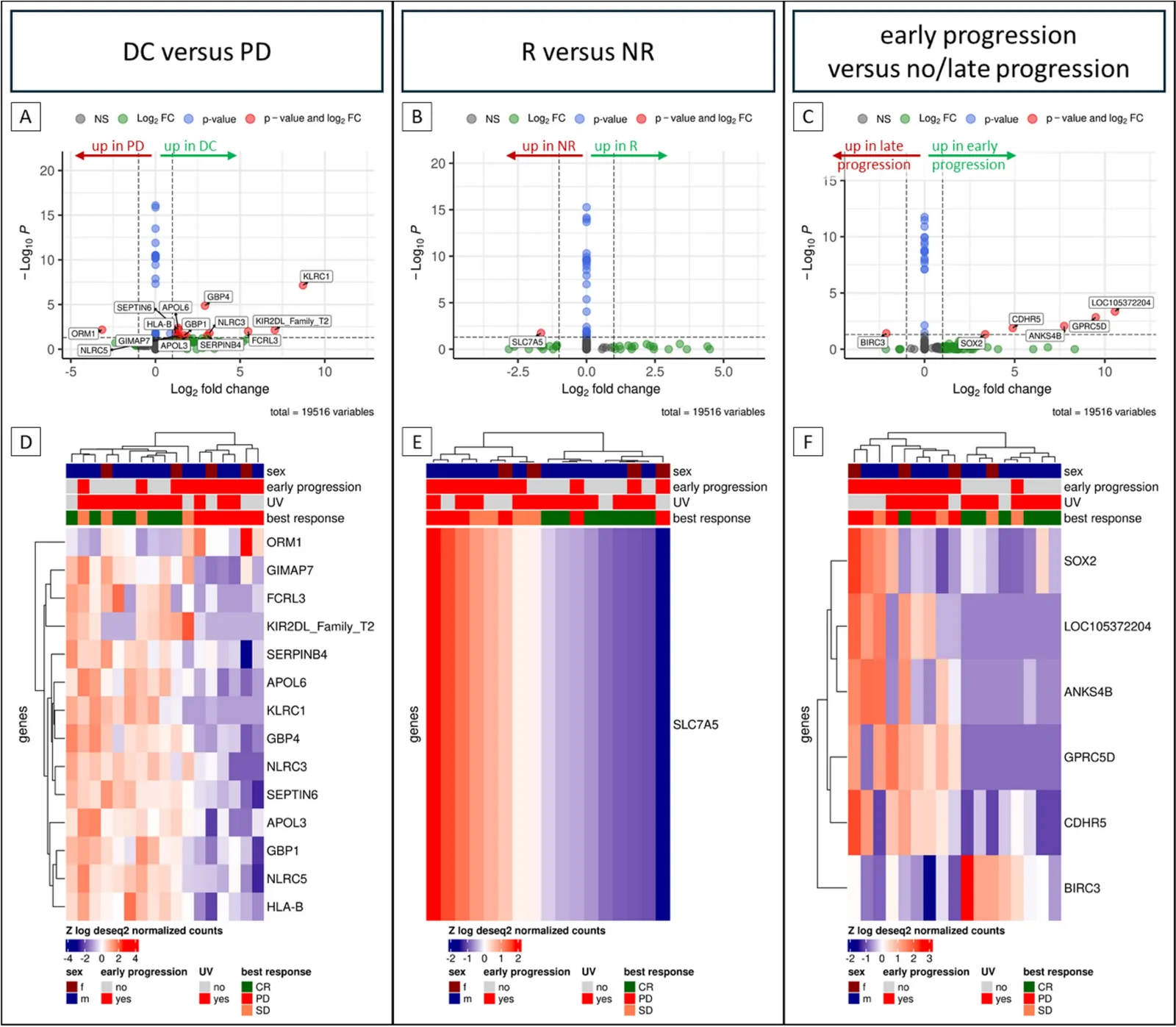
Differential gene expression. Following whole transcriptome analysis, the volcano plots display differentially expressed genes (DEG) in 17 pre-treatment tumor samples based on clinical outcomes ranked by fold change and statistical significance. **A** DC (CR/PR/SD versus PD), positive FC represents upregulation in DC group; **B** Responder (CR/PR) versus Non-Responder (SD/PD), positive FC represents upregulation in R group; **C** Early progression (PFS < 1 year) versus no/late progression (PFS > 1 year), positive FC represents upregulation in early progression group. The corresponding heatmaps show significant DEGs in unsupervised hierarchical clustering; heat represents z-scores of DESeq2 normalized DEGs (D/F/G). *P* values are corrected for multiple testing by the Benjamin-Hochberg procedure. A DEG is considered significant if padj < 0.05 and |logFC| > 1

The most pronounced transcriptional differences were observed between patients with disease control and those with PD (Fig. 1A). In detail, several genes were significantly up-regulated in the DC group under ICI, including APOL3, APOL6, FCRL3, GBP1, GBP4, GIMAP7, HLA-B, KLRC1, NLRC3, NLRC5, SEPTIN6, SERPINB4 and KIR2DL family transcripts, whereas ORM1 showed higher expression in the PD group. Unsupervised hierarchical clustering on the z-scores of these genes resulted in a distinct expression pattern of patients according to clinical response (Fig. 1D), delineating a distinct immune-inflamed transcriptional profile predictive of benefit from ICI therapy. In contrast, the responder versus non-responder comparison revealed only SLC7A5 as DEG (Fig. 1B). The corresponding heatmap illustrates the differential expression of SLC7A5 across individual tumors (Fig. 1E). Similarly, comparison of patients with early progression and those with late or no progression identified only a small set of differentially expressed genes (Fig. 1C). These included SOX2, LOC105372204, ANKS4B, GPRC5D, CDHR5, and BIRC3, which displayed distinct expression patterns across the two progression groups (Fig. 1F). Thus, early progression was associated with a comparatively restricted transcriptional signature rather than the broader immune-related profile observed for disease control.

**Fig. 2:**
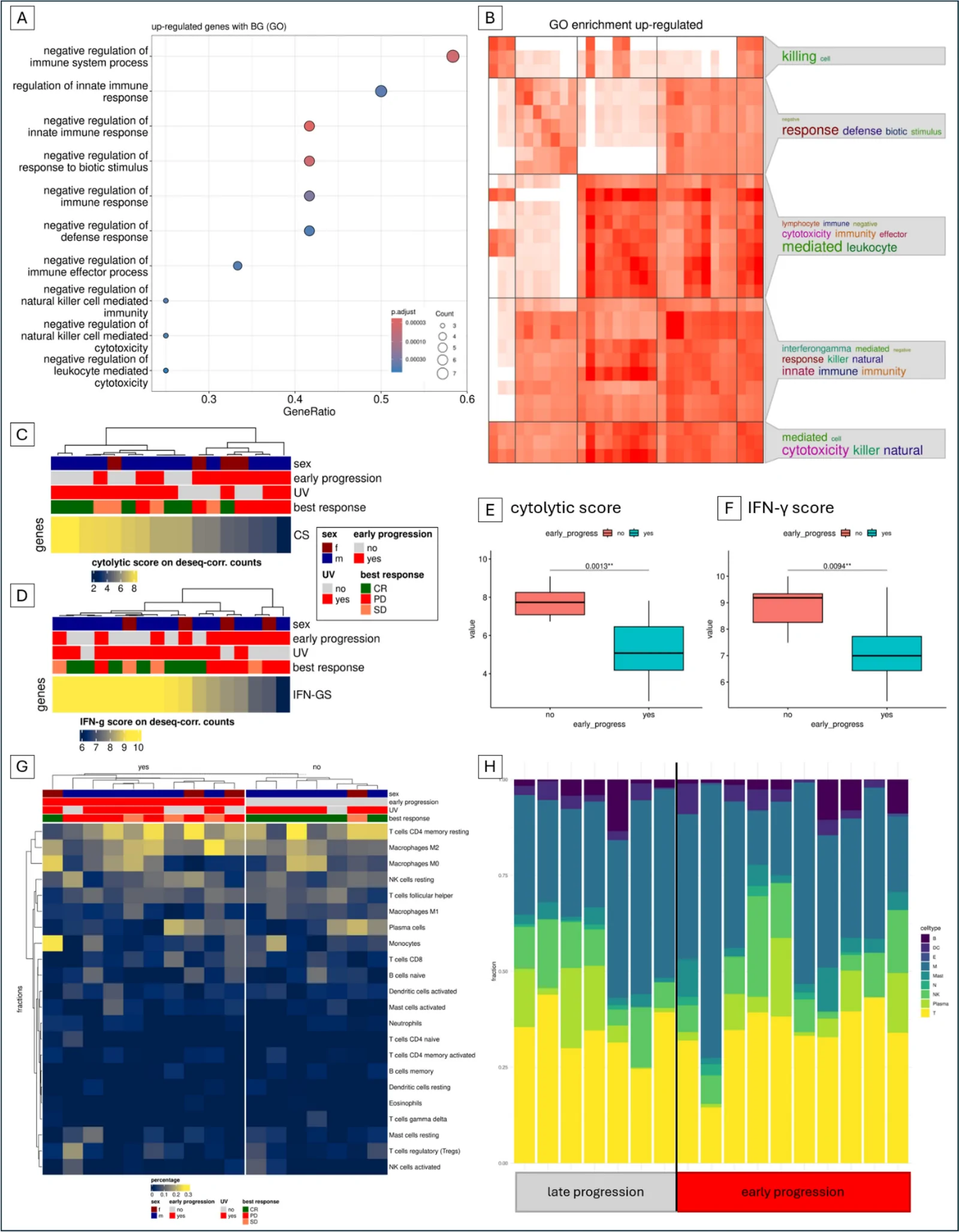
Gene ontology enrichment, immune gene signatures, and CIBERSORTx-based immune cell composition. **A** Gene ontology (GO) enrichment analysis of up-regulated DEGs, showing significantly enriched biological processes. Dot size represents the number of genes associated with each GO term, while color indicates the adjusted *P* value. **B** Heatmap of enriched GO terms illustrating similarities and correlations among related biological processes and revealing distinct functional clusters. **C-F** Annotated heatmaps and dichotomized box plots showing the (**C**, **E**) cytolytic score (CS) and **D**, **F** IFN-γ gene signature (IFN-GS) across 17 pre-treatment cSCC samples. Samples are hierarchically clustered in an unsupervised manner. **E**, **F** A Wilcoxon signed-rank test was applied. **G** CIBERSORTx-derived relative abundance of immune cell populations across pre-treatment tumor samples. Hierarchical supervised clustering according to early progression status. **H** Stacked bar plot summarizing the CIBERSORTx-estimated immune cell composition of individual samples, with immune cell subsets aggregated into major cell-type categories and samples separated into late- and early progression groups. **CS**, cytolytic score; **IFN-GS**, interferon-γ gene signature; **CR**, complete response; **PR**, partial response; **SD**, stable disease; **PD**, progressive disease; **m**, male; **f**, female

Gene ontology (GO) enrichment analysis of genes up-regulated in patients with disease control further indicated enrichment of immune-related biological processes, including regulation of innate and adaptive immune responses, interferon-associated signaling, and T- and NK-cell-mediated cytotoxicity (Fig. 2A/B). Moreover, we showed that immune-related gene signatures, including the previously reported cytolytic (CS) (Fig. 2C, E) and IFN-γ scores (Fig. 2D, F), were elevated among patients with durable clinical responses, supporting their role as indicators of a pre-existing inflamed tumor microenvironment.

To further characterize the immune cellular composition of the tumor microenvironment, CIBERSORTx-based deconvolution was performed. The inferred immune cell fractions revealed marked interpatient heterogeneity in the abundance of different immune cell populations (Fig. 2G). Overall, the immune infiltrate was predominantly composed of T cells, macrophages, and NK cells, with additional contributions from B-cell and plasma-cell populations. When immune cell populations were summarized into broader cell-type categories, the overall composition was largely comparable between patients with early and late/no progression, whereas considerable interindividual variability was observed within both groups (Fig. 2H). To further assess the consistency of these findings, an additional immune cell enrichment analysis was performed using xCell, which provides cell-type enrichment scores (Suppl. Figure 2). Consistent with the CIBERSORTx results, the xCell analysis indicated a heterogeneous immune microenvironment across individual tumors, without an obvious separation of patients according to early progression status. These findings suggest that differences in clinical outcome were not accompanied by a distinct immune cell composition. To complement the transcriptome-based immune deconvolution, the immune cell infiltrate was additionally evaluated semiquantitatively on HE-sections (Fig. 4G–I). Representative tumors showed variable degrees of immune infiltration, ranging from low to intermediate and high infiltration (Fig. 4G–I). Patients achieving disease control showed a tendency toward a higher degree of immune cell infiltration compared with patients with PD, although the distributions overlapped substantially between groups (Fig. 4J).

Given the exploratory nature of the transcriptome-wide analysis and the relatively limited number of robust DEGs across the different clinical comparisons, we next adopted a more targeted approach. We selected genes, which are included into two well-established immuno-oncology panels that are frequently used in the literature to analyze immune-related transcriptional programs in the tumor microenvironment and in the context of immune checkpoint blockade [14]. This strategy was intended to complement the unbiased RNA-seq analysis and to assess whether previously described immune signatures could be reproduced and related to clinical outcome. A list of the selected genes can be found in the supplementary informations.

**Fig. 3:**
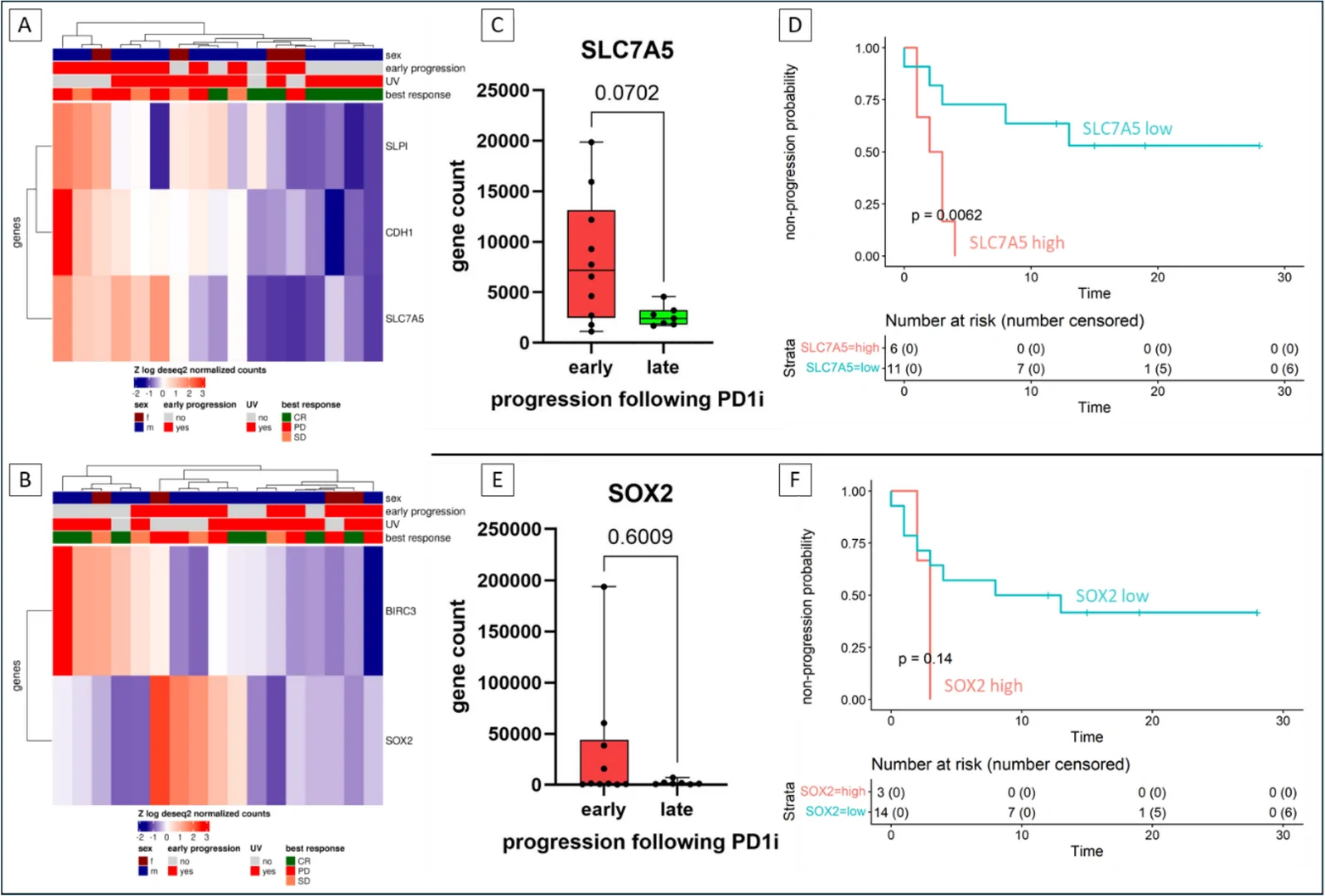
Targeted transcriptional expression analysis. **A**/**B** Heatmaps showing expression of DEGs out of 1291 genes specifically involved in immuno-oncology. **C**/**E** Comparison of SLC7A5 and SOX2 expression between patients with early and late/no progression following immune checkpoint inhibition (PD1i). **D/F** Cutoff values for dichotomization into high- and low-expression groups were determined by receiver operating characteristic (ROC) curve analysis. **D** Kaplan–Meier analysis of non-progression probability according to SLC7A5 expression. **F** Kaplan–Meier analysis according to SOX2 expression. Numbers at risk are shown below the Kaplan–Meier plots. Group comparisons in C and E were performed using an unpaired, nonparametric Mann–Whitney U test. Survival curves in D and F were compared using the log-rank (Mantel–Cox) test. CR, complete response; PR, partial response; SD, stable disease; PD, progressive disease; m, male; f, female

Notably, SLC7A5 and SOX2, both of which had already emerged as candidate genes in the initial transcriptome-wide analysis, were again identified in the more targeted analysis (Fig. 3A and B). Based on their recurrent identification across both approaches and previous reports on these genes to cancer progression, we selected SLC7A5 and SOX2 for further validation and characterization. SLC7A5 showed higher expression in tumors from patients experiencing early progression compared to those with late/no progression, although the difference did not reach statistical significance (*p* = 0.0702; Fig. 3C). Importantly, stratification according to SLC7A5 expression revealed a significantly lower probability of remaining progression free in patients with high SLC7A5 expression (*p* = 0.0062; Fig. 3D). In contrast, SOX2 expression showed a more heterogeneous pattern. While a small subset of patients with PD exhibited particularly high SOX2 expression, this was not reflected in a significant difference at the group level between patients with early and late/no progression (*p* = 0.6009; Fig. 3E). Consistently, SOX2 expression was not significantly associated with progression free outcome (*p* = 0.14; Fig. 3F).

### Protein level validation and correlation analyses

**Fig. 4:**
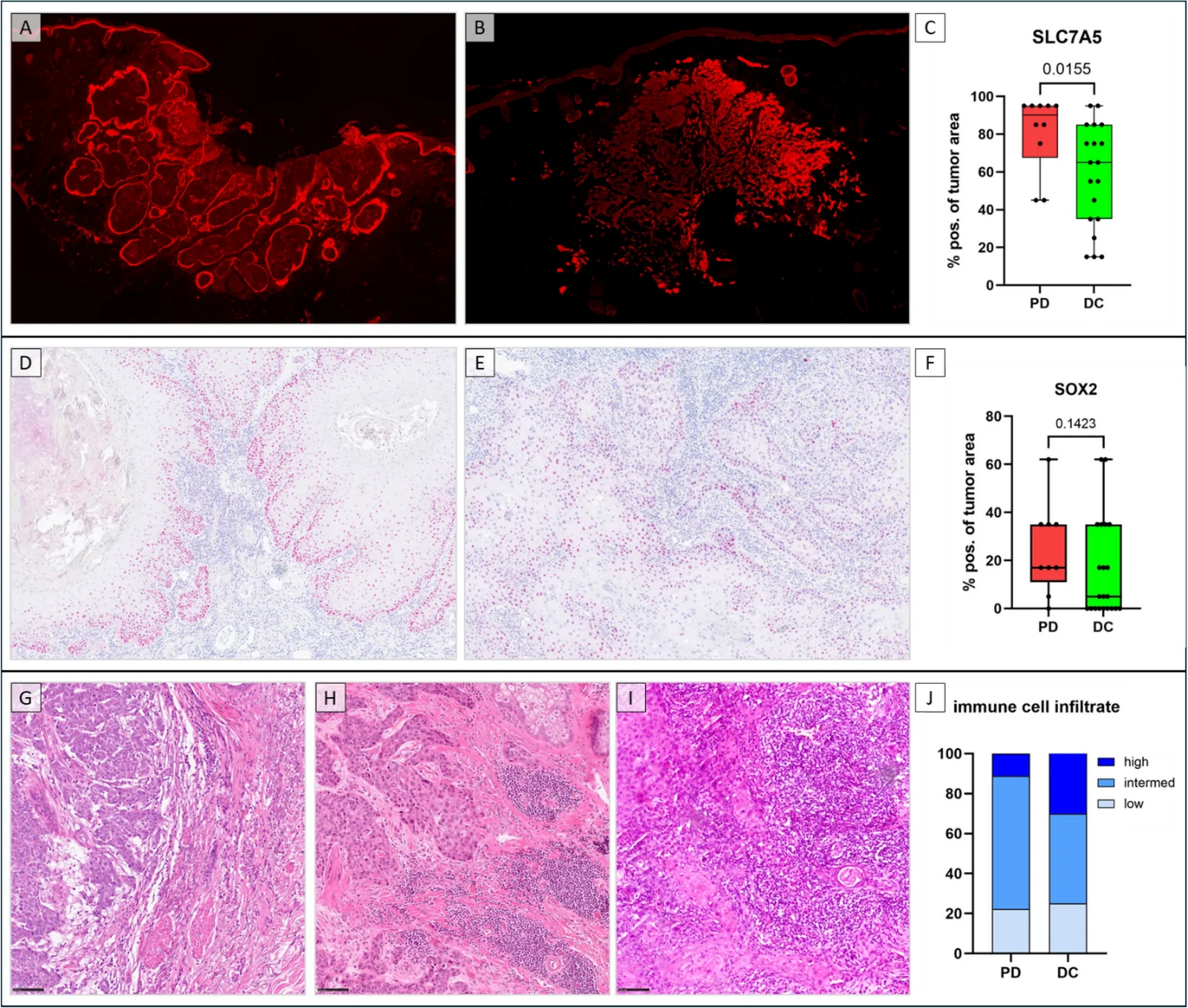
Potential predictive biomarkers at protein level. Representative images of protein expression of SLC7A5 in immunofluorescence (**A/B**) as well as SOX2 (**D/E**) in immunohistochemistry. Box plots presenting percentage of positive tumor area comparing PD versus DC. Group comparisons in C and F were performed using an unpaired, nonparametric Mann–Whitney U test. **C** SLC7A5 (n=22), and SOX2 (n=30). Representative images of low (**G**), intermediate (**H**) and high (**I**) score immune cell infiltrate. Distribution of immune cell infiltrate score for PD versus DC group (**J**, n = 29). PD, progressive disease; DC, disease control

For protein level validation, we expanded the initial transcriptomic cohort by additional patients, as suitable tumor tissue for protein staining was not available from all patients included in the HTG transcriptomic analysis. Immunofluorescent and immunohistochemical staining of cSCC tissue confirmed the expression of SLC7A5 (Fig. 4A/B) and SOX2 (Fig. 4D/E) in tumor cells also at protein level.

Importantly, tumors from non-responder patients and from those with PD exhibited a higher proportion of SLC7A5-positive tumor area (Fig. 4C) and a tendency of higher SOX2 expression (Fig. 4F), thereby corroborating the mRNA expression data.

## Discussion

Clinical trials as well as real-world evidence in advanced cSCC have demonstrated objective response rates of approximately 50% with PD-1 inhibitors such as pembrolizumab and cemiplimab, underscoring their substantial clinical efficacy in this disease setting [4, 5]. As a result, ICI has become firmly established in the routine management of advanced cSCC. However, nearly half of treated patients fail to derive durable benefit, or experience early disease progression, highlighting a critically unmet need to identify predictive biomarkers of response and to better understand the biological mechanisms underlying therapeutic resistance.

In the present study, we performed a transcriptomic comparison of tumor tissue including the surrounding tumor microenvironment (TME) between patients with different clinical response patterns. Tumors from patients achieving disease control displayed a distinct transcriptional profile characterized by upregulation of genes associated with IFN-γ–driven inflammation, enhanced antigen presentation, and the presence of activated cytotoxic and innate immune cell populations. In particular, increased expression of GBP1, GBP4, APOL3, APOL6, HLA-B, NLRC5, KLRC1, GIMAP7, FCRL3, NLRC3, and SEPTIN6 supports the presence of a pre-existing immune-active tumor microenvironment. Several of these genes are associated with interferon-induced responses, antigen processing and presentation, or T- and NK-cell biology, consistent with the GO enrichment analysis demonstrating activation of immune regulatory and cytotoxic pathways. This molecular pattern is a hallmark of immunologically “hot” tumors, which are known to be more susceptible to PD-1 blockade [15]. Notably, similar interferon-associated gene signatures have been linked to responsiveness to immunotherapy in other tumor entities, including melanoma, colorectal, renal and pulmonal cancer, supporting the broader relevance of this immune activation pattern [10, 16–18].

Concurrent elevation of HLA-B and NLRC5, the latter acting as a master regulator of MHC class I transcription, further indicates that tumors from responding patients are highly competent in antigen presentation, enabling efficient recognition by cytotoxic T cells [19]. This is in line with Lee et al., who could show in a spacial transcriptomic approach, that a high antigen presentation niche in cSSC is more frequent in responders [20]. In addition, increased expression of GIMAP7, FCRL3, KLRC1, and KIR2DL family transcripts is consistent with increased representation of lymphocyte-associated transcriptional programs within the tumor microenvironment, particularly involving T- and NK-cell-related immune programs [21–24].

This interpretation was further supported by the analysis of established immune-related transcriptional scores. Both the cytolytic activity score and the IFN-γ-associated gene signature showed higher levels in tumors from patients with more favorable clinical outcomes. These signatures have previously been associated with response to ICI in other malignancies [9, 10, 25, 26]. Together, these findings indicate that response to PD-1 blockade in cSCC is associated with a pre-existing immune-active tumor microenvironment rather than with a single immune marker. Similar relationships between IFN-γ-associated transcriptional programs, cytotoxic immune activity, and immune cell composition have been reported in other tumor entities [27]. However, CIBERSORTx analysis revealed considerable interpatient heterogeneity in the estimated abundance of individual immune cell subsets. Overall, T cells, macrophages, and NK cells represented prominent components of the inferred immune infiltrate, with additional contributions from B-cell and plasma-cell populations. When immune cell subsets were summarized into broader categories, the overall immune cell composition appeared largely comparable between patients with early and late/no progression. Thus, early progression was not associated with a distinct composition of the immune infiltrate detectable by CIBERSORTx. The histopathological evaluation of immune infiltration provided complementary information. Although patients achieving disease control showed a tendency toward a higher degree of immune infiltration than patients with PD, considerable overlap between the groups remained.

We complemented the exploratory whole transcriptome analysis with a more targeted transcriptional approach using genes included into two established panels enriched for immuno-oncology-related genes. This approach reduced the dimensionality of the analysis while allowing focused evaluation of predefined immune- and tumor-associated pathways that have been extensively investigated in the context of cancer immunotherapy. Notably, SLC7A5 (LAT1) and SOX2, which had already emerged as candidate genes in the initial transcriptome-wide analysis, were again identified in the targeted analysis. Elevated expression of these genes was not only associated with early disease progression, as demonstrated by Kaplan–Meier regression, but also reflects biologically relevant tumor-intrinsic programs. These molecules have previously been linked to metabolic adaptation, stemness-associated and survival-promoting pathways [28–31], suggesting that tumors prone to early immune escape may harbor intrinsic resistance mechanisms already present at baseline. Importantly, we were able to detect these targets at the protein level in the majority of cSCC tumor samples, further supporting their biological relevance. Moreover, following semi-quantitative evaluation, increased protein expression of SLC7A5 was observed in non-responders and in patients who developed PD, consistent with our transcriptomic findings. SOX2 protein expression showed also a tendency toward higher expression in patients with PD, although this difference did not reach statistical significance. However, distinct expression patterns were observed. Tumors from patients with PD more frequently displayed a predominant staining pattern at the invasive front.

SLC7A5 is a transmembrane amino acid transporter that mediates the uptake of essential neutral amino acids and is overexpressed in several malignancies, including cSCC [32, 33]. Its expression is induced under hypoxic conditions, which are frequently exacerbated within the tumor microenvironment. Through enhanced import of exogenous amino acids such as leucine, a key driver of mTOR signaling and tumor angiogenesis, SLC7A5 supports tumor cell survival under nutrient- and oxygen-limited conditions, thereby promoting tumor growth and progression [33]. Accordingly, elevated SLC7A5 expression is associated with growth-promoting metabolic conditions. In line with these observations, we detected lower SLC7A5 expression in responding tumors. Importantly, activated T cells are likewise highly dependent on nutrient availability to sustain proliferation and effector function. Experimental work has shown that metabolic competition between malignant cells and tumor-infiltrating T cells can impair antitumor T cell activity and thereby promote tumor progression [34]. It is therefore tempting to speculate that increased SLC7A5-mediated nutrient uptake by tumor cells may contribute to a metabolically competitive microenvironment that compromises T cell function and facilitates resistance to ICI. However, this mechanistic link remains hypothetical and will require functional validation. In parallel, SOX2, a transcription factor and marker of cancer stem cells, has been implicated in tumor-intrinsic resistance mechanisms across multiple cancer types, including cSCC and melanoma [28, 29, 35, 36]. We observed that the nuclear SOX2 protein staining was mainly localized at the tumor-stroma interface of more undifferentiated tumor cells. This is in accordance with Siegle et al., who suggested a critical role for SOX2 in promoting proliferation and cell survival, thus driving rapid SCC growth [36]. Its elevated expression may facilitate early immune escape by promoting survival, metabolic adaptation, and reduced susceptibility to IFN-γ–mediated growth arrest, thereby contributing to early disease progression despite ICI [37].

Several limitations of the present study need to be acknowledged. Most importantly, the transcriptomic analyses were performed in a small exploratory cohort comprising pre-treatment tumor samples from 17 patients. Given the high dimensionality of whole transcriptome profiling relative to the number of patients, individual differential expression findings may be susceptible to sampling variability. Accordingly, the identified genes and signatures should be regarded as hypothesis-generating candidate biomarkers rather than validated predictors of treatment response. Independent validation in substantially larger cohorts will be required.

In summary, our data demonstrate, that clinical benefit from PD-1 blockade in advanced cutaneous squamous cell carcinoma is strongly associated with a pre-existing, IFN-γ–driven, immune-inflamed tumor microenvironment characterized by enhanced antigen presentation and active cytotoxic immune cell engagement. Moreover, early disease progression appears to be driven by tumor-intrinsic resistance programs involving metabolic adaptation and stemness-associated pathways, as reflected by elevated expression of SLC7A5 and SOX2 at transcriptomic and protein level. These findings highlight the combined impact of the immune environment and tumor-intrinsic features on ICI outcomes, supporting the development of integrated biomarkers that consider both aspects. Prospective validation of these results could improve patient stratification and support the development of combination therapies to overcome resistance mechanisms.

## Supplementary Information

Below is the link to the electronic supplementary material.

Supplementary file1 (DOCX 413 KB)

## Data Availability

The data that support the findings of this study are available from the corresponding author, Katrin Schaper-Gerhardt, upon reasonable request.

## Abbreviations

CR: Complete response
cSCC: Cutaneous squamous cell carcinoma
DAPI: 4',6-Diamidino-2-phenylindole
DEG: Differentially expressed gene
DC: Disease control
f: Female
FC: Fold change
FFPE: Formalin fixed paraffin embedded
GO: Gene ontology
ICI: Immune checkpoint inhibition
irAE: Immune-related adverse events
lacSCC: Locally advanced cSCC
m: Male
mcSCC: Metastatic cSCC
NCI CTCAE: National Cancer Institute Common Terminology Criteria for Adverse Events
NA: Not available
NPP: Nuclease protection probes
OS: Overall survival
PR: Partial response
PCR: Polymerase chain reaction
PD-1: Programmed death protein 1
PD: Progressive disease
PFS: Progression free survival
ROC: Receiver operator curve
SD: Stable disease
