# Supplementary material for: Immune and tumor-intrinsic signatures are associated with resistance and response to PD-1 blockade in cutaneous squamous cell carcinoma
Source: Cancer Immunol Immunother. 2026 Oct 30;75(11):264. doi: 10.1007/s00262-026-04610-w (PMC13723579; doi:10.1007/s00262-026-04610-w)
Supplement: Supplementary file 1 [file 262_2026_4610_MOESM1_ESM.docx]

**Supplementary information**

**Suppl. Figure 1: Consort-Diagram**

**Suppl. Figure 2:**

/PR

**Suppl. Figure 2. Principal component analysis (PCA) of pretreatment cSCC transcriptomic profiles.** PCA was performed on normalized gene-expression data to visualize global transcriptional variability across the analyzed tumor samples and to assess potential clustering according to clinical response. Samples are coloured according to response group, with responders (CR/PR) shown in blue and all other response categories shown in red. Each point represents one patient sample and is labelled with the corresponding sample ID. Principal component 1 (PC1) and principal component 2 (PC2) explain 51% and 11% of the total variance, respectively.

**Suppl. Figure 3:**

late progression

early progression

**Suppl. Figure 3. xCell-based immune cell composition.** Stacked bar plot summarizing the xCell-estimated immune cell composition of individual samples, with immune cell subsets aggregated into major cell-type categories and samples separated into late- and early-progression groups. Similar immune cell distributions compared to CIBERSORTx-based immune cell composition.

**Supplementary Table 1: Differential expressed genes**

| **Disease control versus progressive disease** | | |
| --- | --- | --- |
|  |  |  |
| **Gene** | **logFC** | **Adjusted P value** |
| KLRC1 | 8,711 | 7,1456E-08 |
| GBP4 | 2,923 | 1,3591E-05 |
| APOL6 | 1,334 | 4,0587E-03 |
| ORM1 | -3,153 | 6,6059E-03 |
| SEPTIN6 | 1,364 | 6,6059E-03 |
| KIR2DL_Family_T2 | 7,047 | 7,1709E-03 |
| FCRL3 | 5,473 | 9,9437E-03 |
| NLRC3 | 3,189 | 1,5745E-02 |
| HLA-B | 1,187 | 1,7698E-02 |
| GBP1 | 1,709 | 2,8543E-02 |
| SERPINB4 | 3,053 | 3,1547E-02 |
| NLRC5 | 1,388 | 3,3321E-02 |
| GIMAP7 | 1,234 | 3,3405E-02 |
| APOL3 | 1,863 | 3,8002E-02 |
|  |  |  |
| **Responder versus Non-responder** | | |
|  |  |  |
| **Gene** | **logFC** | **Adjusted P value** |
| SLC7A5 | -1,661 | 1,79E-02 |
|  |  |  |
| **Early progression versus no/late progression** | | |
|  |  |  |
| **Gene** | **logFC** | **Adjusted P value** |
| LOC105372204 | 10,557 | 4,5106E-04 |
| GPRC5D | 9,494 | 1,4413E-03 |
| ANKS4B | 7,745 | 8,5311E-03 |
| CDHR5 | 4,891 | 1,3100E-02 |
| BIRC3 | -2,116 | 3,8713E-02 |
| SOX2 | 3,366 | 4,6417E-02 |
